# High Maternal Serum Estradiol Levels Induce Dyslipidemia in Human Newborns via a Hepatic HMGCR Estrogen Response Element

**DOI:** 10.1038/srep10086

**Published:** 2015-05-11

**Authors:** Ye Meng, Ping-Ping Lv, Guo-Lian Ding, Tian-Tian Yu, Ye Liu, Yan Shen, Xiao-Ling Hu, Xian-Hua Lin, Shen Tian, Min Lv, Yang Song, Meng-Xi Guo, Zhang-Hong Ke, Hong Xu, Jian-Zhong Sheng, Feng-Tao Shi, He-Feng Huang

**Affiliations:** 1 International Peace Maternity and Child Health Hospital, School of Medicine, Shanghai Jiao Tong University, Shanghai 200030, China; 2 Key Laboratory of Reproductive Genetics, Ministry of Education (Zhejiang University), Hangzhou, Zhejiang 310006, China; 3 Department of Pathophysiology, School of Medicine, Zhejiang University, Hangzhou, Zhejiang 310058, China; 4 Institute of Embryo-Fetal Original Adult Disease Affiliated to Shanghai Jiao Tong University School of Medicine, Shanghai 200030, China

## Abstract

While the intrauterine environment is essential for the health of offspring, the impact of high maternal serum estradiol (E_2_) on lipid metabolism in offspring and the mechanisms are unknown. We found that ovarian stimulation (OS) could result in high E_2_ levels in women throughout pregnancy. Strikingly, their newborns showed elevated total cholesterol (TC) and low-density lipoprotein cholesterol (LDL-C) levels that were positively related with E_2_ in newborns. *In vitro*, E_2_ dose-dependently stimulated TC and LDL-C secretion, and increased expression of the cholesterol synthesis rate-limiting enzyme 3-hydroxy-3-methylglutaryl-CoA reductase (HMGCR) in HepG2 cells and mouse fetal hepatocytes. *In vivo*, high maternal E_2_ was detected and fetal livers also showed significantly higher HMGCR expression in an OS mouse model. Notably, an estrogen response element (ERE) was identified in the HMGCR promoter, indicating that high maternal serum E_2_ could up-regulate HMGCR expression in fetal hepatocytes via an ERE that in turn induces elevated levels of TC and LDL-C in offspring. Conclusion: OS can induce a high maternal E_2_ environment, which up-regulates HMGCR expression in fetal hepatocytes via an ERE in the promoter, and induces elevated levels of TC and LDL-C in newborns that may be related to increased risk of metabolic disease in adulthood.

Adverse experiences early in life can profoundly impact the risk of developing diseases later in life^1^,^2^,^3^,^4^. The “developmental origin of health and disease” (DOHaD) theory posits that the health of an individual is closely related to their health status early in life^5^. Current research on the fetal origin of adult diseases is now more extensive and in-depth, and an increasing number of adverse factors, such as early nutritional deficiencies, chemical exposure and the mother’s emotional state during pregnancy have been identified^6^. Moreover, an increasing number of human and animal studies have related the gestational endocrine environment to long-term effects in offspring. For example, abnormal gestational thyroid hormone concentrations may affect the neural development of the fetus^7^, while maternal exposure to bisphenol A leads to hallmarks of allergic lung inflammation in adult offspring^8^. Perinatal testosterone exposure could affect the development and sexual behavior of female rats^9^.

Over the past three decades assisted reproductive technology (ART), especially *in vitro* fertilization-embryo transfer (IVF-ET), has been used to treat infertility, with the number of children resulting from these procedures now exceeding 5 million worldwide. However, the long term impacts on health of offspring associated with these technologies remain unknown^10^. Ovarian stimulation (OS), which is a standard component of IVF-ET, can place the fetus in a supraphysiological maternal estradiol (E_2_) environment^11^. Our previous study showed that in newborns conceived by OS, a high maternal E_2_ environment in the first trimester was correlated with an increased risk of low birth weight (LBW) as well as infants who were small for gestational age (SGA)^12^. Importantly, LBW has been found to be related to a cluster of adult metabolic diseases^13^,^14^,^15^,^16^,^17^. A continuous, graded relationship exists between lipid levels and risk of metabolic disorders^18^. In addition to the effect on birth weight as reported in our previous study^12^, our present study aims to know whether high maternal serum E_2_ could directly cause dyslipidemia in offspring, which can increase the risk of adult chronic diseases.

In this study we examined maternal E_2_ levels during different stages of pregnancy after OS, determined the lipid profile of the newborns conceived by OS, and analyzed the correlation between maternal E_2_ and newborn lipid levels. Furthermore, we investigated the expression of the cholesterol synthesis rate-limiting enzyme 3-hydroxy-3-methylglutaryl-CoA reductase (HMGCR) in human hepatoma (HepG2) cells and mouse fetal hepatocytes treated with E_2_ *in vitro* as well as in fetal livers from a high intrauterine E_2_ mouse model *in vivo*. Finally, we identified an estrogen response element (ERE) in the HMGCR promoter that may be mechanistically involved with these observed effects.

## Results

### High maternal E_2_ levels during pregnancy after OS

**Figure 1:**
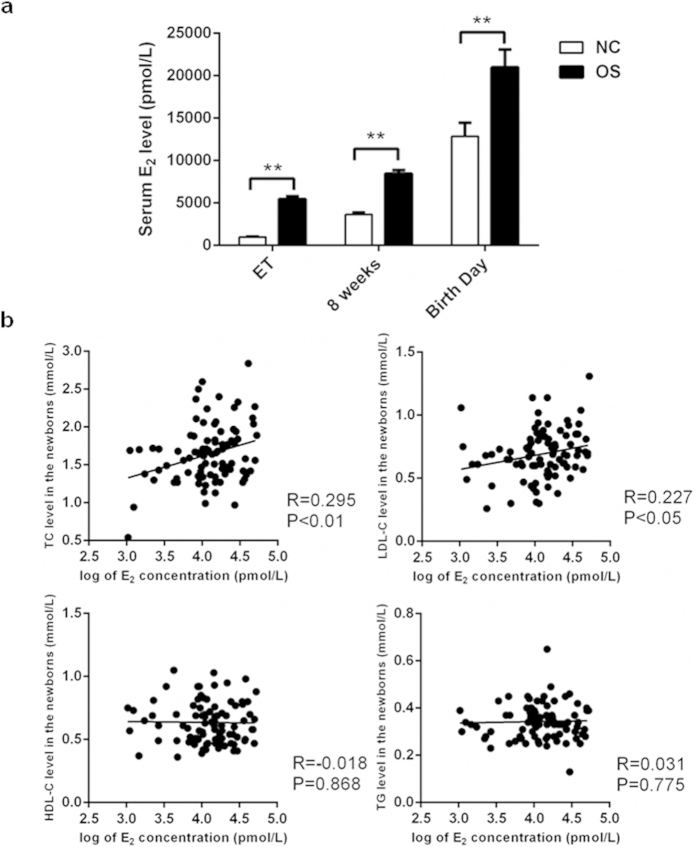
Dyslipidemia in the newborns conceived by OS were correlated to high maternal E_2_ levels throughout pregnancy. (**a**) The levels of E_2_ at different stages throughout pregnancy. The mean serum levels of E_2_ in the OS group at different stages throughout pregnancy (at ET day, n = 89; 8 weeks, n = 89; Birth day, n = 44) and in the NC group (at the same time point as ET day, n = 62; 8 weeks, n = 151; Birth day, n = 44). Data were presented as mean ± SEM. *P < 0.05 and **P < 0.01 compared with the corresponding control group, respectively. (**b**) The correlation between lipid levels and the E_2_ concentrations in the umbilical cord blood of newborns. TC and LDL-C levels were positively correlated with the E_2_ concentrations in the 44 newborns (R = 0.295, *P* < 0.01; R = 0.227, *P* < 0.05; respectively). In contrast, the amounts of HDL-C and TG had no correlation with the E_2_ levels in the 44 newborns (R = −0.018, *P* = 0.868; R = 0.031, *P* = 0.775; respectively). OS: ovarian stimulation; E_2_: estradiol; ET: embryo transfer day; 8 weeks: 8 weeks of gestation; NC: natural conception; TC: total cholesterol; LDL-C: low-density lipoprotein cholesterol; HDL-C: high-density lipoprotein cholesterol; TG: triglyceride.

We examined the serum E_2_ levels of IVF (*in vitro* fertilization) patients and NC (natural conception) women on the days of ET (embryo transfer) as well as at 8 weeks of gestation, and the serum E_2_ levels of umbilical cord blood on the birth day. The mean serum E_2_ levels in the OS group were all significantly higher than those in the NC group at different stages throughout pregnancy (5466.65 ± 320.28 vs. 975.29 ± 65.12 pmol/L on the ET day or the same time point, 8460.08 ± 433.50 vs. 3661.63 ± 222.06 pmol/L at 8 weeks, and 20980.68 ± 2093.62 vs. 12870.67 ± 1586.82 pmol/L on the birth day, respectively; *P* < 0.01) (Fig. 1a).

### Dyslipidemia in newborns of the OS group and correlation with maternal E_2_

Maternal body mass index, systolic pressure, diastolic pressure, heart rate, and gestational age as well as serum TC (total cholesterol), LDL-C (low density lipoprotein), high-density lipoprotein cholesterol (HDL-C), and triglyceride (TG) levels during late pregnancy were comparable in the 44 newborns from the OS group and the 44 newborns from the NC group (Table 1). There was no significant difference in maternal serum lipid levels, body mass index, blood pressure, heart rate, neonatal gender or birth weight between the OS and NC groups. Although the expected maternal age was slightly higher in the OS group, there was no relationship between maternal age and lipid levels in the progeny (Supplementary Fig. S1).

Lipid concentrations in umbilical cord blood were significantly different between the two groups. The levels of TC and LDL-C in the OS group were significantly higher than those in the NC group (1.78 ± 0.47 vs. 1.50 ± 0.22 mmol/L, *P* < 0.01 and 0.74 ± 0.18 vs. 0.54 ± 0.21 mmol/L, *P* < 0.05, respectively), while the level of HDL-C was significantly lower in newborns conceived by OS compared to those who were conceived naturally (0.58 ± 0.12 vs. 0.69 ± 0.18 mmol/L, respectively; *P* < 0.01). There was no significant difference in TG levels between the two groups (0.35 ± 0.07 vs. 0.33 ± 0.07 mmol/L; *P* = 0.12) (Table 1).

As shown in Fig. 1b, TC and LDL-C levels were positively correlated with the E_2_ concentrations in umbilical cord blood (TC: R = 0.295, *P* < 0.01; LDL-C: R = 0.227, *P* < 0.05). However, the levels of HDL-C and TG did not correlate with newborn E_2_ levels (HDL-C, R = −0.018, *P* = 0.868; TG, R = 0.031, *P* = 0.775).

### Elevated lipid secretion and HMGCR expression in HepG2 cells treated with E_2_

**Figure 2:**
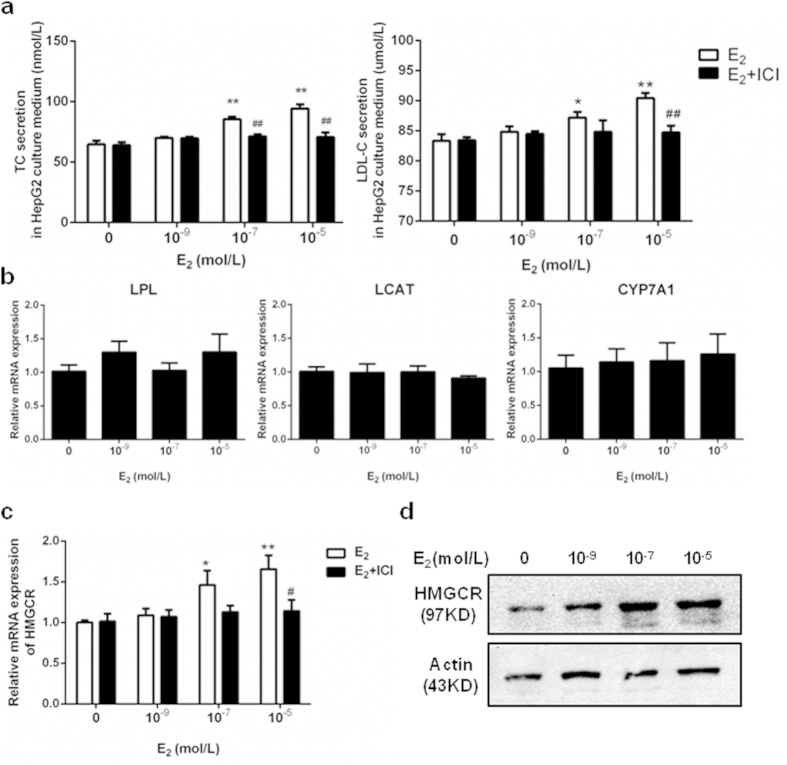
E_2_ increased the lipid secretion levels and HMGCR expression in HepG2 cells in a dose-dependent manner, effects attenuated in the presence of ICI, an ER antagonist. (**a**) The secretion levels of TC and LDL-C in HepG2 cells after 24 hours treatment with different doses of E_2_, or co-treatment with E_2_ and ER antagonist ICI (10^−5^ mol/L). (**b**) The LPL, LCAT, and CYP7A1 mRNA expression in HepG2 cells after 24 hours treatment with E_2_ in a dose-dependent manner. (**c**) The HMGCR mRNA expression in HepG2 cells after 24 hours treatment with E_2_ in a dose-dependent manner or co-treatment with E_2_ and ICI (10^−5^ mol/L). (**d**) The HMGCR protein expression in HepG2 cells after 24 hours treatment with E_2_ in a dose-dependent manner. The experiments were repeated three times and data were presented as Mean ± SEM. *P < 0.05 and **P < 0.01 compared with the corresponding control group, respectively. #*P* < 0.05 and ##*P* < 0.01 compared with the value in the E_2_-treated group, respectively. E_2_: estradiol; TC: total cholesterol; LDL-C: low-density lipoprotein cholesterol; ICI: ICI 182,780; ER: estrogen receptor; LPL: lipoprotein lipase; LCAT: lecithin cholesterol acyl transferase; CYP7A1: cholesterol 7α-hydroxylase; HMGCR: 3-hydroxy-3-methylglutaryl-CoA reductase.

E_2_ stimulated TC and LDL-C secretion in HepG2 cells in a dose-dependent manner (Fig. 2a), and the increased secretion levels were blocked after treatment with by ICI 182,780 (ICI, 10^−5^M), which is a selective estrogen receptor (ER) antagonist^19^. As shown in Fig. 2b, E_2_ had no effect on lipoprotein lipase (LPL), lecithin-cholesterol acyltransferase (LCAT), or cholesterol 7α-hydroxylase (CYP7A1) mRNA expression. In contrast, E_2_ increased the amount of HMGCR mRNA (Fig. 2c) and protein (Fig. 2d) expression in HepG2 cells in a dose-dependent manner. Moreover, the increases in HMGCR mRNA levels were blocked by treatment with ICI (Fig. 2c).

### Upregulation of HMGCR in E_2_-treated primary fetal hepatocytes and fetal livers **of OS mice**

**Figure 3:**
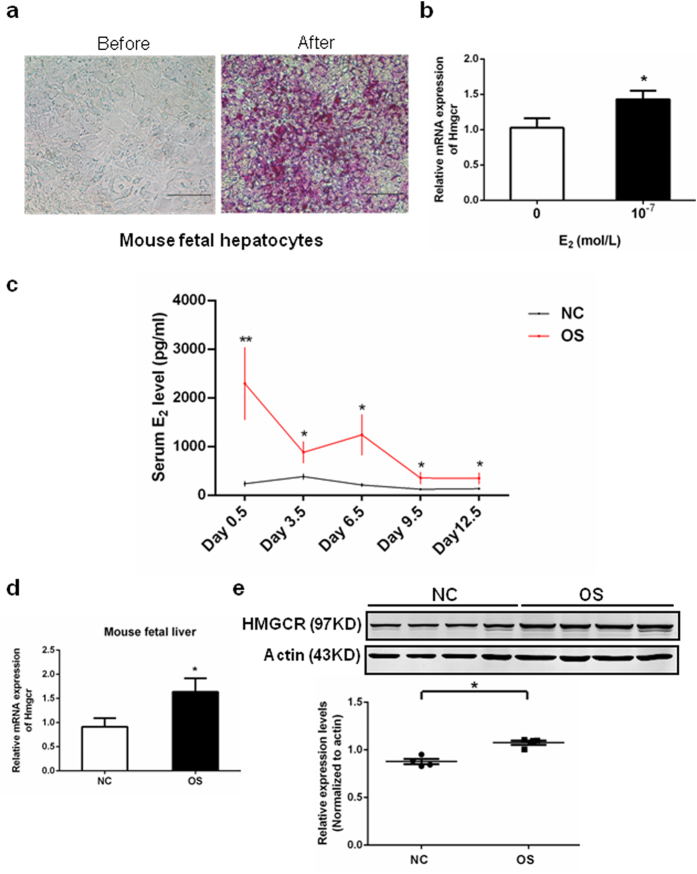
The HMGCR mRNA expression was stimulated by E_2_ in mouse fetal hepatocytes *in vitro* or fetal livers of OS mouse *in vivo*. (**a**) Mouse fetal hepatocytes were identified by PAS staining. Scale bar, 500 μm. (**b**) The HMGCR mRNA expression in primary mouse fetal hepatocytes after 24 hours treatment with E_2_ (10^−7^ mol/L). At the experiment of (**a**) and (**b**) about ten to twenty fetuses of day 12.5 from one pregnant mouse were used to make the primary hepatocyte cultures every time. Their livers were collected and pooled together. The experiment was repeated three times and the fetuses from 3 pregnant mice were used in total. (**c**) The level of E_2_ during pregnancy in NC or OS mice at five time points (day 0.5: NC n = 7, OS n = 5; day 3.5: NC n = 5, OS n = 5; day 6.5: NC n = 5, OS n = 5; day 9.5: NC n = 9, OS n = 5; day 12.5: NC n = 8, OS n = 5; the sample numbers were varied because it was difficult to get the blood of mouse at some time points). (**d**) The HMGCR mRNA expression in fetal livers of NC or OS mice (n = 10 per group). (**e**) The HMGCR protein expression in fetal livers of OS or NC mice (n = 4 per group). The *top* panel shows immunoblots result and the *bottom* panel shows the relative protein expression levels of HMGCR normalized to those of β-actin. Data were presented as Means ± SEM. *P < 0.05 and **P < 0.01 compared with the corresponding control group, respectively. HMGCR: 3-hydroxy-3-methylglutaryl-CoA reductase; E_2_: estradiol; OS: ovarian stimulation; PAS: periodic acid schiff; NC: natural conception.

We next cultured primary culture of fetal hepatocytes in order to study the effects of E_2_ on HMGCR gene expression. Periodic acid Schiff (PAS) stain is a traditional method for identifying hepatocytes^20^ as shown in Fig. 3a. PAS staining was used to identify mouse fetal hepatocytes, but not all of hepatocytes could be dyed. In our experiments, PAS positive cells were more than 90% in the cell population and the following experimental results should not be influenced a lot. HMGCR expression in primary mouse fetal hepatocytes markedly increased after treatment with E_2_ (10^−7^ mol/L) for 24 hours (Fig. 3b). In the OS mouse model, serum E_2_ levels were significantly higher during pregnancy (Fig. 3c), and HMGCR mRNA and protein expression were also significantly increased in fetal livers on pregnancy day 12.5 (Fig. 3d,e).

### Identification of an ERE in the HMGCR promoter

**Figure 4:**
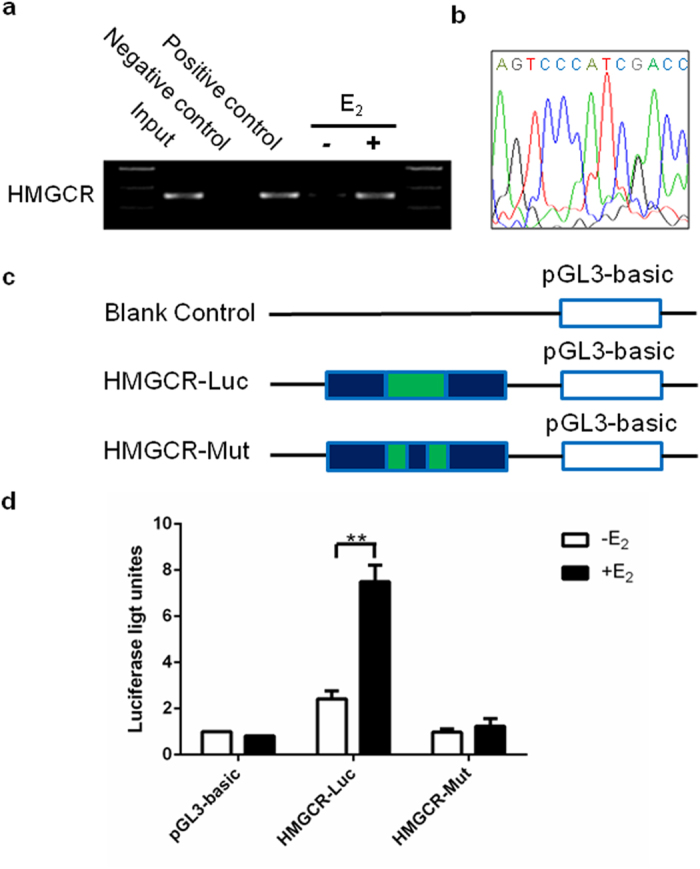
A functional ERE was identified within the HMGCR promoter. (**a**) ChIP analysis was performed using anti-ERα or anti-RNA Polymerase II antibody to ascertain the existence of the ERE in the promoter of the HMGCR gene. The PCR results showed that one fragment containing the putative ERE could be precipitated after treatment of HepG2 with E_2_ (10^−7^ mol/L) for 24 hours. (**b**) The pulled down band was excised from the gel and sequenced. (**c**) Schematic diagram of luciferase reporter constructs. Blank control: pGL3-basic plasmid; HMGCR-Luc: pGL3-basic plasmid with the putative ERE-like sequence insert; HMGCR-Mut: pGL3-basic plasmid with the mutative ERE-like sequence insert. (**d**) Luciferase activities of three report systems in the presence or absence of E_2_ (10^−7^ mol/L) were compared with each other. The experiments were repeated three times and data were presented as Means ± SEM. **P < 0.01 compared with the value in non-E_2_ treated control group. ERE: estrogen response element; HMGCR: 3-hydroxy-3-methylglutaryl-CoA reductase; ChIP: chromatin immunoprecipitation; E_2_: estradiol.

ER is a transcription factor that binds to a specific DNA sequence known as the estrogen response element (ERE: GGTCAnnnTGACC) with high affinity and transactivates gene expression in response to estradiol^21^. High-scoring ERE-like sequences in the HMGCR gene promoter were identified by bioinformatics analysis. To further confirm that the HMGCR gene is regulated directly by estrogen through an ERE, ChIP (chromatin immunoprecipitation) analysis was performed. The results showed that one fragment containing the putative ERE in 13 nucleotides (AGTCCcatCGACC) was captured by ChIP after administration of E_2_ (Fig. 4a,b). To determine whether the putative ERE plays a functional role in estrogen-dependent transcriptional activation, we transfected HepG2 cells with three luciferase reporter constructs (HMGCR-Luc, HMGCR-Mut or blank control) and measured their activation after treatment with E_2_ (Fig. 4c). Only the construct containing the putative ERE (AGTCCcatCGACC) was activated by E_2_, whereas cells carrying the construct in which the element was mutated (CCCAGcctCTCCG) were unaffected (Fig. 4d). These results indicate that a functional ERE motif exists in the promoter fragment of the HMGCR gene that mediates estrogen-dependent HMGCR expression.

## Discussion

ART first emerged in 1978 and continues to evolve today. However, increasing evidence has indicated that ART may predispose individuals to increased incidence of metabolic alterations, such as obesity and subclinical hypothyroidism as well as elevated blood pressure, fasting glucose and triglycerides^22^. However, to date no study has investigated the correlation of high maternal serum E_2_ with metabolic risks in IVF offspring or the underlying mechanisms responsible for such risks. Identification of specific molecules that are affected by high E_2_ will facilitate the development of novel therapies and may improve current IVF clinical approaches. In our previous study, we found that OS resulted in increased E_2_ concentrations during the first trimester that were correlated with increased risks of LBW^12^. A larger sample size was included and the difference of birth weight between the two groups was modest but reaches statistical significance. Our present study aims to know whether high maternal serum E_2_ could directly cause dyslipidemia in offspring and that may be related to increased risk of metabolic disease in adulthood. Thus, we just chose the specimens of normal birth weight, and the birth weight was comparable between the two groups. We report here for the first time that high E_2_ levels induced by OS can persist throughout the entire pregnancy period and may directly influence the lipid metabolism of offspring.

E_2_ produced in pregnant women can be distributed via the bloodstream through the placenta and into the fetal blood supply, which may affect fetal metabolism and health^23^. Thus, safety concerns for both mothers and offspring are heightened due to high serum E_2_ levels resulting from OS^24^,^25^. In our study, we found that OS increased E_2_ concentrations not only before and during implantation, but also after implantation, during the first trimester^12^, and even throughout the gestation period. Up until birth, E_2_ levels remained dramatically higher in the OS-treated group compared to the controls. In early pregnancy, estradiol is secreted by the corpus luteum. Ovarian stimulation could enlarge multifollicular ovaries and lead to a very high plasma levels of estradiol because the formation of multiple corpora lutea after oocyte retrieval. Once placenta is formed, estradiol is mainly secreted by placenta. Ovarian stimulation could also affect placental development in both animal and human studies^26^,^27^ and may stimulate estradiol synthesis signaling pathways that lead to the excessive synthesis of estradiol. Thus, the estradiol level was still high in the pregnant mother even after cessation of ovarian stimulation. Our result suggests that the fetuses of OS-treated patients were exposed to a high E_2_ environment throughout the pregnancies.

Moreover, the levels of TC and LDL-C, which are the lipids mainly synthesized in the liver^28^, were also positively correlated with E_2_ levels in newborns. Consistent with the clinical observations, TC and LDL-C can be induced by E_2_ in HepG2 cells *in vitro*. In addition, the effects of E_2_ are blocked by treating cells with the selective ER antagonist ICI 182,780, which competes with estrogen for binding to the ER with high affinity and disrupts ER nucleocytoplasmic shuttling by preventing its dimerization^29^,^30^. This inhibitor also decreases ER expression by reducing the protein half-life^31^. There were no significant differences of the maternal serum lipid levels between the OS and NC groups at the third trimester. However, in Brizzi’s study, maternal lipid levels were significantly increased on day 15 after hCG (human chorionicgonadotropin) administration, but they did not continue the measurement further^32^. High serum E_2_ levels continued throughout pregnancy. During this period, embryo/fetus is in the progress of rapid cell differentiation and organ formation, and might be more sensitive to the high E_2_ environment. In addition, the maternal body has better self-regulation ability generally than the fetus and the liver is well-developed. Although exposed to high E_2_ levels, their blood lipid levels might be self-regulated to normal levels before childbirth. Thus, the levels of TC and LDL-C were not increased in maternal serum at the third trimester in the presence of high estradiol while significantly increased in the offspring.

Several important enzymes are involved in lipid metabolism, including LPL, LCAT, CYP7A1, and HMGCR. LPL hydrolyzes circulating triglycerides and liberates free fatty acids^33^, while LCAT esterifies free cholesterol primarily at the surface of the HDL particle, which results in the migration of cholesteryl ester molecules migrating to the inner core of the HDL particle^34^. The enzyme CYP7A1 catalyzes the initial step in cholesterol catabolism and bile acid synthesis^35^. HMGCR is the rate-limiting step in cholesterol biosynthesis^36^ and its activity can be inhibited by several cholesterol-lowering drugs such as rosuvastatin and simvastatin^37^. We found that E_2_ had no effect on the expression of other enzymes, but significantly up-regulated HMGCR, which was confirmed by the inhibition seen after treatment with ICI. Interestingly, HMGCR expression was also clearly increased in fetal livers of an OS mouse model, and significantly high maternal E_2_ levels were observed during pregnancy. This result indicates that liver damage resulting from high E_2_ levels can occur as early as the in utero phase. *In vitro* cultures also confirmed the effect of high E_2_ on HMGCR expression in fetal mouse hepatocytes, providing a probable mechanism for how high maternal serum E_2_ levels can directly influence cholesterol synthesis and induce fetal lipometabolic disturbances. As such, our *in vivo* and *in vitro* data confirmed that exposure to high E_2_ environment can result in the increased expression of HMGCR in fetal hepatocytes and lead to dyslipidemia in offspring.

This study identified a functional ERE within the HMGCR promoter for the first time. Estrogen exerts its effects by directly binding to ER, which then homodimerizes and interacts with ERE to stimulate the transcription of target genes. The palindromic sequence of the ERE in the HMGCR promoter is similar to the canonical ERE^37^. However, most E_2_-regulated genes also have imperfect, variant EREs^38^. Our results suggest that HMGCR expression is under the direct control of E_2_ via the ERE in the HMGCR gene promoter.

In summary, our findings highlight a novel link between high maternal serum E_2_ levels and metabolic risks in IVF offspring. An environment high in E_2_ may upregulate expression of the cholesterol biosynthesis rate-limiting enzyme HMGCR in fetal hepatocytes via an ERE in the promoter and further induce high TC and LDL-C levels in newborns (see Supplementary Fig. S2, schematic of proposed mechanism). Importantly, these changes may further increase the risk of developing metabolic diseases later in life. Our results suggest that after OS, the ET should be canceled if maternal E_2_ levels are extremely high in order to avoid fetal exposure to an environment that is high in estrogen. For those children who have experienced exposure to high E_2_ environment, their lipid levels should be monitored so that early intervention can be undertaken to reduce the risk of adult metabolic disease.

## Patients and Methods

### Patients and sample collection

All experiments were performed in accordance with relevant guidelines and regulations. Ethical approval for this project was granted by the Ethics Committee of the School of Medicine, Zhejiang University and was registered in the Chinese Clinical Trial Registry (ChicCTR-OCC-14004682). A written informed consent was obtained from each subject before sample collection. Characteristics and cycle parameters of patients were obtained from databases. Blood samples of 240 women with singleton conceptions at 8 weeks of gestation were obtained, including 89 singleton conceptions after OS, which is the routine procedure of IVF, and 151 singletons after NC. The serum E_2_ levels at ET day of the 89 singleton conceptions after OS and 62 singletons after NC at the same time point were obtained from databases. Serum E_2_ concentrations were all measured with Roche cobas immunoassay (Roche Diagnostics, Mannheim, Germany). Umbilical cord blood specimens of 44 healthy singletons conceived by OS and 44 naturally conceived singletons born during the same period were collected to detect E_2_ and lipid levels by Beijing North Institute of Biological Technology (Beijing, China). The blood pressure, heart rate and serum lipid levels of patients at the third trimester time point were collected while the birth weight and gender ratio of offspring were comparable in the two groups. The correlation between the umbilical E_2_ and lipid levels were calculated, with missing data excluded.

During all OS groups, ovarian stimulation was performed by the standard long or short protocols as previously described^39^. Only data from singletons born alive after the 28th week of gestation were included in the analysis. Births from OS group were excluded if the women had received donated oocytes or sperm, or applied preimplantation genetic diagnosis. The patients with obstetric complications, such as pregnancy induced hypertension, gestational diabetes mellitus, and placenta previa, were excluded as well.

### Establishment of OS animal model

All animal protocols were reviewed and approved by the Zhejiang University Animal Care and Use Committee. The imprinting control region (ICR) mice were housed in 12/12 hours light/dark cycle at 25 ± 0.5 °C and 50–60% of humidity, and fed with a standard diet and water ad libitum. ICR females received intraperitoneal (i.p.) injection of 10 IU PMSG (pregnant mare serum gonadotropin; Pregnyl, Organon, the Netherlands) and followed by an injection (i.p.) of 10 IU hCG (Pregnyl) 46–48 hours after administration of PMSG, then mated with ICR males and checked for a vaginal plug the morning following mating. The day that the vaginal plug was first seen was designated as day 0.5. The blood samples of pregnant mice at day 0.5, day 3.5, day 6.5, day 9.5 and day 12.5 were collected in OS and NC group to detect E_2_ levels by Beijing North Institute of Biological Technology (Beijing, China), 5–10 cases per group at each time point (day 0.5: NC n = 7, OS n = 5; day 3.5: NC n = 5, OS n = 5; day 6.5: NC n = 5, OS n = 5; day 9.5: NC n = 9, OS n = 5; day 12.5: NC n = 8, OS n = 5; the sample numbers were varied because it was difficult to get the blood of mouse at some time points). After the blood collection for E_2_ measurement, the mice were killed and the fetal livers of day 12.5 were obtained from 10 different pregnant mice each group.

### Cell culture and ELISA assays

HepG2 cell line (1 × 10^4^ /ml) from human hepatoma was maintained in phenol red DMEM high-glucose medium (Gibco-BRL, Gaithersburg, MD) supplemented with 10% fetal bovine serum (FBS, Gibco-BRL) and 100 U/ml penicillin and streptomycin (Gibco-BRL) in 95% CO_2_ at 37 °C. When the cells were confluent to 40–50%, the medium was replaced by phenol red free DMEM (Gibco-BRL) supplemented with 1% charcoal-stripped fetal bovine serum (Gibco-BRL), and 17β-estradiol (E_2_; 7.14 nmol/L; Sigma-Aldrich, St. Louis, MO) were added into the culture medium at different concentrations for 24 hours. We measured the concentrations of TC and LDL-C in culture media of HepG2 cells using an ELISA kit (R&D, Minneapolis, MN) according to the manufacturer’s instruction. The experiments were repeated three times.

The mouse fetal liver was formed at day 10.5 of gestation^40^ while it was difficult to collect fetal livers at that time. We obtained the fetal livers at day 12.5 of gestation when the levels of serum E_2_ were still significantly higher in OS pregnant mice at this time point. Primary hepatocytes from 12.5-day-old NC fetal mice were isolated by collagenase (Sigma-Aldrich). Fetuses from one pregnant mouse, about ten to twenty fetuses, were used to make the primary hepatocyte cultures every time. The experiments were repeated three times. Because primary hepatocyte cells require rich nutrition, cells (1 × 10^4^ /ml) were cultured in DMEM high-glucose medium supplemented with 20% FBS and incubated in 5% CO_2_ at 37 °C. A PAS stain was used for the identification of hepatocytes.

### Periodic acid schiff (PAS) staining

The PAS technique was used to determine the presence of insoluble stored glycogen content in the cells. Briefly, cultured primary fetal hepatocytes were fixed with 4% paraformaldehyde at room temperature for 15 minutes. They were then oxidized with 1% periodic acid for 5 minutes, rinsed with distilled water for 5 minutes and treated with Schiff’s reagent for 10–15 minutes. After rinsing in distilled water for 5 minutes, dishes were counter-stained with Mayer’s hematoxylin for 30 seconds and observed under light microscope.

### RT-PCR and Quantitative real-time PCR analysis

We extracted total RNA from scraped cells or tissues using the Trizol reagents (Takara, Dalian, China) and reverse-transcribed first-strand cDNA according to the protocol of PrimeScript™ RT reagent Kit (Takara). The PCR was performed in a thermal cycler (PTC-200 DNA Engine; MJ Research, Waltham, MA) and RT-PCR products were visualized in 1% agarose gels. Real-Time PCR analysis was carried out in an Applied Biosystems 7900 Fast (Applied Biosystems, Carlsbad, CA). Data were analyzed by the comparative threshold cycle method with normalization to glyceraldehyde-3-phosphate dehydrogenase (GAPDH). Changes after treatments were recorded as fold differences from values in untreated controls. The HMGCR, LPL, LCAT and CYP7A1 mRNA expression in HepG2 cells, as well as the HMGCR mRNA expression in primary mouse fetal hepatocytes were examined. The experiments were repeated three times and in each experiment, measurements were made in duplicate. Besides, the HMGCR mRNA expression in fetal livers of OS or NC mice (n = 10 per group) was examined and in each experiment, measurements were made in duplicate. The primers used for Quantitative real-time PCR and PCR were listed in Supplementary Table S1.

### Western blotting analysis

First the samples were homogenized in 1xRIPA buffer [10 mmol/L Tris-HCl (pH 8.0), 140 mmol/L NaCl, 1 mmol/L EDTA (pH 8.0), 0.5 mmol/L EGTA, 1% Triton X-100, 0.1% SDS, 0.1% sodium deoxycholate] containing protease inhibitors (Sigma-Aldrich). The homogenate was incubated on ice for 40 minutes and centrifuged at 15,000 g for 15 minutes. The protein concentration in supernatant was determined by Bradford assay (Bio-Rad Laboratories, Hercules, CA). Samples at 50 μg/lane were separated on a 10% SDS-PAGE gel. The separated samples were transferred to nitrocellulose membranes and exposed to rabbit anti-HMGCR antibody (1:200; Santa Cruz Biotechnology, Santa Cruz, CA) and rabbit anti-β-actin antibody (1:5000; Santa Cruz Biotechnology) for overnight at 4 °C. After washing, the membranes were incubated with the secondary antibody, a horseradish peroxidase-conjugated goat anti-rabbit IgG (1:10,000, Santa Cruz Biotechnology) for 1 hour at room temperature, and visualized with enhanced chemiluminescence reagents (Santa Cruz Biotechnology). The experiment was repeated three times.

### Bioinformatics analysis and ChIP assay

The regulatory sequence analysis tools ( http://rsat.ulb.ac.be/rsat/) were used to analyze the promoter sequence of HMGCR to find high scoring ERE-like sequences. After treatment with 10^−7^ mol/L E_2_ for 24 hours, HepG2 cells were cross-linked and processed according to the Millipore EZ-ChIP Assay Kit protocol (Millipore, Temecula, CA). A relevant antibody from Millipore was used for immunoprecipitation: mouse anti-human ERα ChIP validated antibody, mouse anti-human RNA Polymerase II antibody (positive control), or mouse IgG (negative control). PCR analysis for HMGCR was carried out using the primers listed in Supplementary Table S1 and the PCR products were analyzed using agarose electrophoresis. The pulled down band was excised from gel and sequenced by Invitrogen (Shanghai, China).

### Luciferase reporter assay

Two DNA fragments for the putative (AGTCCcatCGACC) or mutated (CCCAGcctCTCCG) ERE-like sequence synthesized by Gene Copoeia (Guangzhou, China) were digested with XhoI and KpnI (Thermo Fisher Scientific, Waltham, MA) and ligated into pGL3-basic plasmid (Promega, Madison, Wisconsin) to construct the HMGCR promoter luciferase reporter systems: HMGCR-Luc and HMGCR-Mut. HepG2 cells were seeded in 6-well tissue culture plates and grown in phenol red-free medium DMEM supplemented with 1% charcoal/dextran-treated FBS. Cells were co-transfected with HMGCR promoter luciferase reporter plasmid and pRL-TK reporter plasmid (cDNA encoding Renilla luciferase; Promega) by using the Fugene HP transfection reagent (Roche Applied Science, Basel, Switzerland). After 24 hours transfection, HepG2 cells were treated with 10^−7^ mol/L E_2_ for an additional 24 hours period. Luciferase activities in cell lysates were measured using the dual-luciferase reporter assay system (Promega) according to the manufacturer’s instructions. Luciferase values were normalized to the Renilla luciferase activity.

### Statistical analysis

Either the 2-tailed student’s t test or ANOVA was used to evaluate statistical significances of continuous parametric data, as appropriate. The chi-square test was used to compare categorical data. Pearson correlation analysis was adopted to investigate the correlation between lipid and E_2_ levels in the newborns. All statistical analyses were performed in SPSS 16.0. Results were reported as means ± SEM and were considered significantly different from each other at *P* < 0.05.

## Author Contributions

Y.M. mainly carried out the study and prepared the manuscript; the co-first author P.P.L. contributed to the animal model study; G.L.D helped designed the experiment; T.T.Y. did part of western study and helped draw graphs; Y.L. had contribution to design the experiment; Y.S. was in charge of cell culture; X.L.H., X.H.L. and S.T. collected the clinical data; M.L. and Y.S. did the statistics; M.X.G. hepled draw graphs; Z.H.K. and H.X. had contribution to real-time PCR and western study; J.Z.S. analyzed data; F.T.S. helped summarize the data and write the manuscript; H.F.H. planned and supervisored the study. All authors have approved the final version and have nothing to disclose.

## Additional Information

**How to cite this article**: Meng, Y. *et al.* High Maternal Serum Estradiol Levels Induce Dyslipidemia in Human Newborns via a Hepatic HMGCR Estrogen Response Element. *Sci. Rep.* **5**, 10086; doi: 10.1038/srep10086 (2015).

## Supplementary Material

Supplementary Information

## Figures and Tables

**Table 1:** Characteristics and lipid profile of the mothers and newborns in NC and OS groups.

**Variable**	**NC (n = 44)**	**OS (n = 44)**	**P value**
**Mothers**			
Age, years	28.48 ± 3.07	32.39 ± 3.10	<0.01
Body mass index, kg/m^2^	27.06 ± 2.99	27.64 ± 2.38	0.29
Systolic pressure, mmHg	114.11 ± 9.76	114.30 ± 9.74	0.40
Diastolic pressure, mmHg	71.56 ± 6.95	72.76 ± 6.56	0.33
Heart rate, rpm	86.43 ± 7.89	85.35 ± 9.89	0.23
Gestational age, weeks	38.57 ± 1.09	38.40 ± 1.10	0.14
Total cholesterol, mmol/L	6.65 ± 1.10	6.76 ± 1.33	0.29
LDL-C, mmol/L	3.13 ± 0.98	2.92 ± 0.89	0.23
HDL-C, mmol/L	1.76 ± 0.36	1.74 ± 0.33	0.34
Triglyceride, mmol/L	3.47 ± 1.07	3.84 ± 1.44	0.23

**Newborns**			
Gender (male), %	61.36	54.55	0.52
Birth weight, kg	3.43 ± 0.35	3.33 ± 0.52	0.43
Total cholesterol, mmol/L	1.50 ± 0.22	1.78 ± 0.47	<0.01
LDL-C, mmol/L	0.54 ± 0.21	0.74 ± 0.18	<0.05
HDL-C, mmol/L	0.69 ± 0.18	0.58 ± 0.12	<0.01
Triglyceride, mmol/L	0.33 ± 0.07	0.35 ± 0.07	0.12

Results were described as means ± SEM and were considered significantly different from each other at *P* < 0.05. P values for comparison of the gender ratio between the two groups were from chi-square test, and P values for comparison of other variables were from 2-tailed student’s t test. Abbreviations: NC, natural conception; OS, ovarian stimulation; LDL-C, low-density lipoprotein; HDL-C, high-density lipoprotein.
