# Supplementary material for: High Maternal Serum Estradiol Levels Induce Dyslipidemia in Human Newborns via a Hepatic HMGCR Estrogen Response Element
Source: Sci Rep. 2015 May 11;5:10086. doi: 10.1038/srep10086 (PMC4426719; doi:10.1038/srep10086)
Supplement: Supplementary Information [file srep10086-s1.pdf]

Supplemental Materials

Title: High Maternal Serum Estradiol Levels Induce Dyslipidemia in Human Newborns via a Hepatic HMGCR Estrogen Response Element

Authors: Ye Meng, Ping-Ping Lv, Guo-Lian Ding, Tian-Tian Yu, Ye Liu, Yan Shen, Xiao-Ling Hu, Xian-Hua Lin, Shen Tian, Min Lv, Yang Song, Meng-Xi Guo, Zhang-Hong Ke, Hong Xu, Jian-Zhong Sheng, Feng-Tao Shi, He-Feng Huang

Contents:

Number of supplemental tables: 1.

Number of supplemental figures: 2.

Table S1 Nucleotide sequences of primers used for quantitative Real-time PCR and PCR

Gene Name	Accession Number	Sequences (5'->3')		Length
Human HMGCR	NM_000859.2	Forward	TGAGGGCTCCTTCCGCTCCG	78bp
		Reverse	ACTAGAGGCCACCGAACCCCG	
Human CYP7A1	NM_000780.3	Forward	TCTTACAAGGCGGGACACAC	166bp
		Reverse	GTTCTCGTGCCTCAAGCTCT	
Human LCAT	NM_000229.1	Forward	CCCCATGGCAGTGGGTGACG	148bp
		Reverse	CAGCCGGGCACGAGGATGAC	
Human LPL	NM_000237.2	Forward	GTGGACTGGCTGTCACGGGC	168bp
		Reverse	GCCAGCAGCATGGGCTCCAA	
Human GAPDH	NM_002046.5	Forward	CAGGGCTGCTTTTAACTCTGG	102bp
		Reverse	TGGGTGGAATCATATTGGAACA	
Mouse Hmgcr	NM_008255.2	Forward	CCACGCAGCAAACATTGTCA	199bp
		Reverse	GCAGGCTTGCTGAGGTAGAA	
Mouse Gapdh	NM_001289726.1	Forward	TGACGTGCCGCCTGGAGAAA	98bp
		Reverse	AGTGTAGCCCAAGATGCCCTTCAG	
Human HMGCR (ChIP)	NC_000005.10	Forward	ACGGAGCCTTAGCTGCCTTC	174bp
		Reverse	GCTCACACTCCTCAGCCCTT	

Abbreviations: HMGCR: 3-hydroxy-3-methylglutaryl-CoA reductase; CYP7A1: cholesterol 7α-hydroxylase; LCAT: lecithin-cholesterol acyltransferase; LPL: lipoprotein lipase, GAPDH: glyceraldehyde-3-phosphate dehydrogenase.

Figure S1

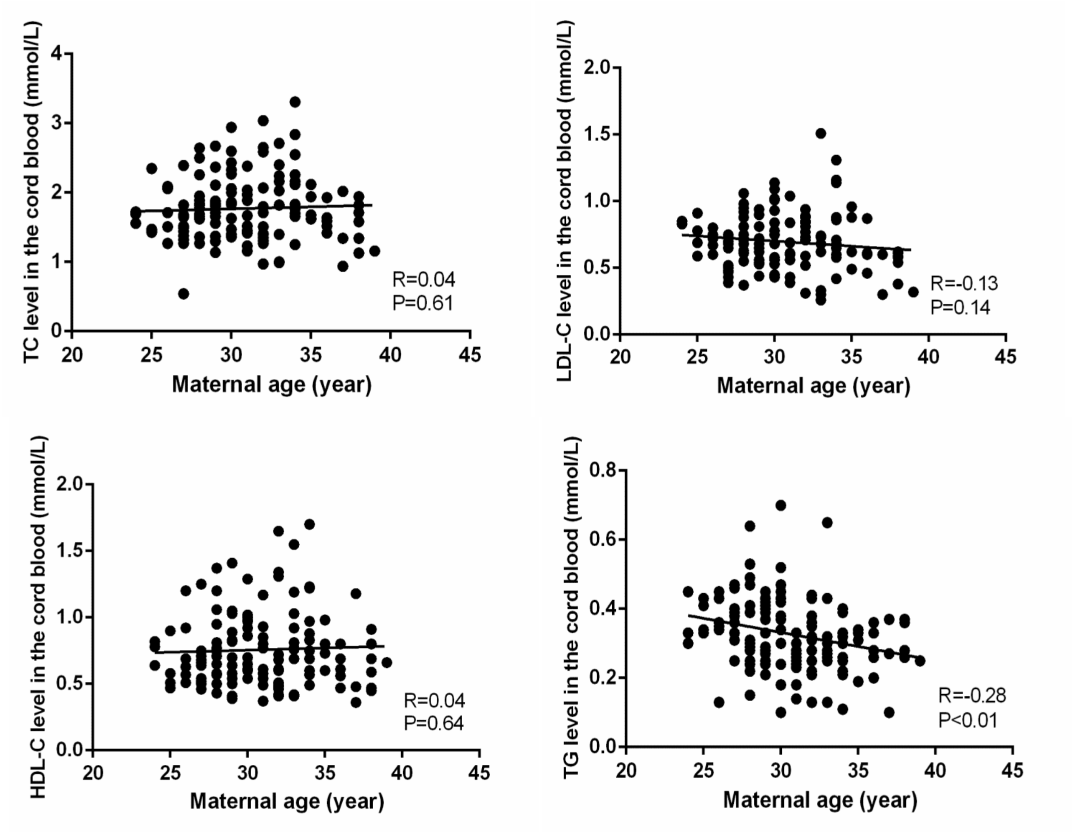

Figure S1. The correlation between lipid levels and the maternal age in the umbilical cord blood of newborns.

The amounts of TC, LDL-C, HDL-C and TG had no correlation with the maternal age in the 44 newborns (R =0.04, *P* = 0.61; R = -0.13, *P* =0.14; R =0.04, *P* = 0.64; R = -0.28, *P*<0.01; respectively). TC: total cholesterol; LDL-C: low-density lipoprotein cholesterol; HDL-C: high-density lipoprotein cholesterol; TG: triglyceride.

Figure S2

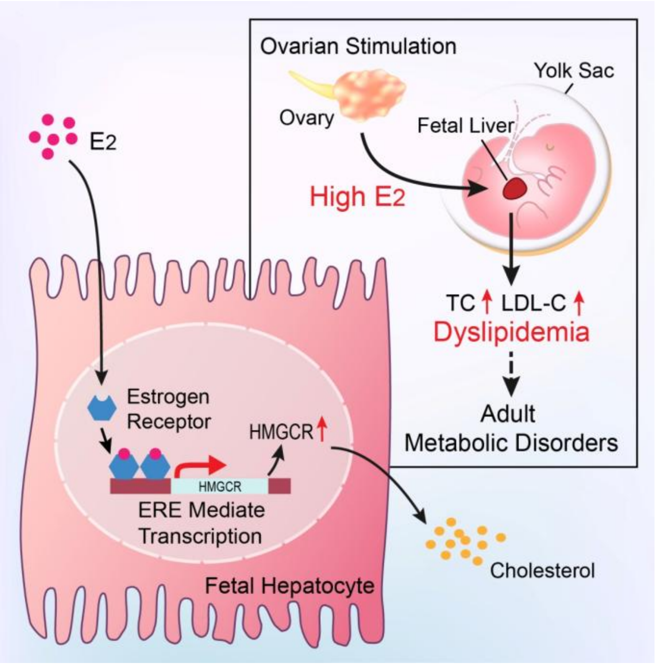

Figure S2. Hypothetical schematic representation of dyslipidemia induced by high E_2 in human newborns.

OS at IVF-ET procedure can induce maternal high E_2 throughout pregnancy. Intrauterine high E_2 can up-regulate the cholesterol synthesis rate-limiting enzyme HMGCR expression in fetal hepatocytes via an ERE in the promoter and induces elevated levels of TC and LDL-C levels in offspring that may be related to increased risk of metabolic diseases in adulthood. OS: ovarian stimulation; E_2: estradiol; IVF-ET: *in vitro* fertilization and embryo transfer; HMGCR: 3-hydroxy-3-methylglutaryl-CoA reductase; ERE: estrogen response element; TC: total cholesterol; LDL-C: low-density lipoprotein cholesterol.
